# Comparison of the Magnetic and Structural Properties of MnFePSi Microwires and MnFePSi Bulk Alloy

**DOI:** 10.3390/ma17081874

**Published:** 2024-04-18

**Authors:** Mohamed Salaheldeen, Valentina Zhukova, James Rosero, Daniel Salazar, Mihail Ipatov, Arcady Zhukov

**Affiliations:** 1 Department of Polymers and Advanced Materials, Faculty of Chemistry, University of the Basque Country, UPV/EHU, 20018 San Sebastián, Spain; valentina.zhukova@ehu.es; 2 Department of Applied Physics I, EIG, University of the Basque Country, UPV/EHU, 20018 San Sebastián, Spain; 3 Physics Department, Faculty of Science, Sohag University, Sohag 82524, Egypt; 4 EHU Quantum Center, University of the Basque Country, UPV/EHU, 20018 San Sebastián, Spain; 5 BCMaterials, Basque Center for Materials, Applications and Nanostructures, 48940 Leioa, Spain; james.rosero@bcmaterials.net (J.R.); daniel.salazar@bcmaterials.net (D.S.); 6 Servicios Generales de Investigación (SGIker), 48080 Bilbao, Spain; mihail.ipatov@ehu.es; 7 IKERBASQUE, Basque Foundation for Science, 48011 Bilbao, Spain

**Keywords:** MnFePSi alloys, glass-coated microwires, magnetic field, Taylor–Ulitovsky technique, coercivity

## Abstract

We provide comparative studies of the structural, morphological, microstructural, and magnetic properties of MnFePSi-glass-coated microwires (MnFePSi-GCMWs) and bulk MnFePSi at different temperatures and magnetic fields. The structure of MnFePSi GCMWs prepared by the Taylor–Ulitovsky method consists of the main Fe_2_P phase and secondary impurities phases of Mn_5_Si_3_ and Fe_3_Si, as confirmed by XRD analysis. Additionally, a notable reduction in the average grain size from 24 µm for the bulk sample to 36 nm for the glass-coated microwire sample is observed. The analysis of magnetic properties of MnFePSi-glass-coated microwires shows different magnetic behavior as compared to the bulk MnFePSi. High coercivity (450 Oe) and remanence (0.32) are observed for MnFePSi-GCMWs compared to low coercivity and remanent magnetization observed for bulk MnFePSi alloy. In addition, large irreversibility at low temperatures is observed in the thermal dependence of magnetization of microwires. Meanwhile, the bulk sample shows regular ferromagnetic behavior, where the field cooling and field heating magnetic curves show a monotonic increase by decreasing the temperature. The notable separation between field cooling and field heating curves of MnFePSi-GCMWs is seen for the applied field at 1 kOe. Also, the M/M_5K_ vs. T for MNFePSi-GCMWs shows a notable sensitivity at a low magnetic field compared to a very noisy magnetic signal for bulk alloy. The common features for both MnFePSi samples are high Curie temperatures above 400 K. From the experimental results, we can deduce the substantial effect of drawing and quenching involved in the preparation of glass-coated MnFePSi microwires in modification of the microstructure and magnetic properties as compared to the same bulk alloy. The provided studies prove the suitability of the Taylor–Ulitovsky method for the preparation of MnFePSi-glass-coated microwires.

## 1. Introduction

Many technological applications, including sensors, electronic security surveillance, microelectronics, medicine, the automotive and aerospace industries, energy harvesting and conversion, home entertainment, electrical engineering, magnetic recording, magnetic memories, etc., have a high demand for magnetic functional materials with tunable magnetic properties and reduced dimensions [1,2,3,4,5,6,7,8,9].

The following characteristics of magnetic functional materials are in high demand: a large magnetoimpedance effect, GMI, magnetocaloric effect, MCE, the Hall effect, energy harvesting, magnetoresistance effects (AMR, GMR, TMR), high magnetic permeability, etc. [9,10,11,12,13,14]. The pricing and properties of magnetic materials have an impact on the cost and performance of sensors and devices.

Some of the above-mentioned properties, such as excellent magnetic softness, high GMI effect, low dimensionality suitable for various industrial applications, and high mechanical and corrosion properties can be obtained in amorphous materials prepared by rapid melt-quenching method [9,12,13,14]. Additionally, rapid melt-quenching technology is generally rather fast and inexpensive. Therefore, rapid melt quenching was also successfully used for the preparation of crystalline and metastable materials with improved properties [9,12,13,14].

MnFePSi alloys have emerged as a prominent and swiftly advancing category of magnetocaloric materials, drawing considerable attention in recent years due to their exceptional magnetic and structural properties [15,16,17,18]. This alloy stands out for its distinctive electronic structure, endowing it with remarkable attributes such as a high magnetocaloric effect (MCE) and substantial saturation magnetization [9,18,19,20]. These features make it a compelling candidate for various applications, as they play a crucial role in enhancing the efficiency of magnetic refrigeration systems and solid-state cooling devices.

The (Mn,Fe)_2_(P,Si)-based materials, including MnFePSi, hold a particularly promising position among the magnetocaloric materials. This is primarily because they offer an ideal combination of characteristics for practical applications, including the utilization of low-cost starting materials and environmentally friendly properties [9,18,19,20,21,22,23,24,25,26]. These materials hold the potential to revolutionize the field of refrigeration and cooling technologies, reducing the environmental impact of conventional cooling methods.

Furthermore, as we move towards an era of miniaturization and micro-scale technology, the development of MCE devices based on small-size MnFePSi particles, wires, ribbons, films, bi- and multilayers, and pillars becomes increasingly significant [15,16,17,18,19,20,21,22,23,24,25,26]. These compact and versatile forms of MnFePSi alloys open the door to a variety of technological applications, ranging from compact cooling devices in electronic components to portable cooling solutions for medical and industrial settings. The adaptability of these materials demonstrates their versatility and potential impact across a wide range of industries.

In light of these factors, the fabrication of thin wires from brittle MnFePSi alloy represents an intriguing research and development route. Such an approach holds substantial promise in terms of practical applications of MCE, potentially leading to the creation of innovative and efficient cooling solutions in various fields [23,26,27]. As we explore the possibilities offered by MnFePSi alloys and their derivatives, we pave the way for a future where sustainable and efficient cooling technologies become more accessible and prevalent. The choice of glass-coating microwire form is due to their wide applications, especially in sensing technology [9,11,12,28,29,30,31,32,33]. Such microwires, often just a few micrometers in diameter, consist of a metallic nucleus coated with a thin insulating glass shell [9,12,28,29,30,31,32,33,34,35,36]. This distinct structure confers several advantages to glass-coated microwires, prompting extensive research and application in various industrial sectors [7,9,11,12,37]. The metallic nucleus material choice is determined by the applications of the microwires. Thus, MnFePSi-based microwires can open many applications that are not used for the bulk material or thin film physical forms. In addition, the glass shell provides several advanced features. It protects the metallic nucleus, i.e., MnFePSi, mechanically, preserving its integrity even in tough situations. Furthermore, the insulating glass coating allows us to avoid electrical short-circuits, enhances corrosion resistance, and provides biocompatibility [7,9,12,38]. During the microwire fabrication process, the thickness of the glass coating can be carefully adjusted, allowing it to be customized to meet the particular sensing requirements. The metallic nucleus diameters, d, can be varied from 0.1 to 100 µm. The Taylor–Ulitovsky method is described in detail elsewhere [9,12,29,36,39]. Recently, several successful attempts have been reported to fabricate glass-coated microwires from Heusler alloy or granular alloys exhibiting MCE or GMR effects [9,34].

In this paper, we provide results on a primary investigation of the morphological, structural, and magnetic properties of MnFePSi metallic alloy in two main forms “bulk” and “glass-coated microwires” to investigate the direct effect of the drawing casting and the glass-coating layer on the magnetic and structural properties of MnFePSi alloy.

## 2. Materials and Methods

The fabrication process of the samples entails a bifurcated procedure, starting with the preparation of MnFePSi in bulk form. This initial stage requires a specific concentration of their constituents, namely Mn (99.5%), P (99.5%), FeP (98%) and Si (99.999%). The primary step involves alloying an ingot via arc melting, conducted within a controlled argon environment. An augmentation of 5 weight percent of Mn and P was deliberately introduced into the mixture to compensate for the Mn and P loss incurred during the arc-melting process. Once the MnFePSi alloy ingot was prepared, we proceeded to use the Taylor–Ulitovsky method to prepare MnFePSi-glass-coated microwires [9,12,30,36,39]. Briefly, the preparation method consists of melting the produced metal ingot (typically a few g) with a high-frequency inductor (often 350–500 kHz) within a Pyrex glass tube. After that, the softened glass is shaped into a capillary and is caught by a rotating receiving spool [9,12,29,36,40,41]. The molten metallic alloy then fills the glass capillary, forming a microwire with a metallic nucleus fully covered with a continuous, thin, and flexible glass coating. In this manufacturing technique, a stream of cooling water is used to achieve a high enough quenching rate of a composite metallic microwire covered with an insulating glass coating [9,12,29,30,36,37].

It is worth noting that this fabrication technique was developed in 60 s for non-magnetic alloys [41]. Subsequently, this method was used for the preparation of amorphous ferromagnetic glass-coated microwires [9,12,29,30,36,37,42]. Great attention has been paid to the preparation and studies of magnetic microwires with amorphous structures owing to their outstanding soft magnetic properties, high GMI effect, or magnetic bistability [9,12,29,36,37,39]. The peculiarity of such a fabrication method is that simultaneous rapid quenching of the metallic alloys and glass coating is the source of rather high internal stresses [9,12,29,30,36,42,43,44]. Recently, this method has been used for the preparation of magnetic microwires with crystalline structures, such as Heusler or granular alloys [9,40,43,45,46,47].

The microstructure, morphology and composition of the produced MnFePSi-glass-coated microwires and bulk MnFePSi alloys were examined using a Hitachi TM3000 scanning electron microscope (SEM) (Tokyo, Japan) equipped with an energy-dispersive spectrometry (EDX) apparatus. Furthermore, a BRUKER X-ray diffractometer (D8 Advance, Bruker AXS GmbH, Karlsruhe, Germany) was utilized to execute Cu Kα (λ = 1.54 Å) radiation for their structural investigation. Magnetic properties of MnFePSi samples have been studied by the Physical Property Measurement System (PPMS, Quantum Design Inc., San Diego, CA, USA). The axial magnetization curves were measured over a broad temperature range (5–300 K) and magnetic field (1–20 kOe). The easy axial direction of magnetization, *M*, is expected due to the shape magnetic anisotropy. Furthermore, magnetic fields, *H*, up to 90 kOe were used.

## 3. Results and Discussion

### 3.1. Morphological and Microstructural Properties of MnFePSi Samples

**Figure 1:**
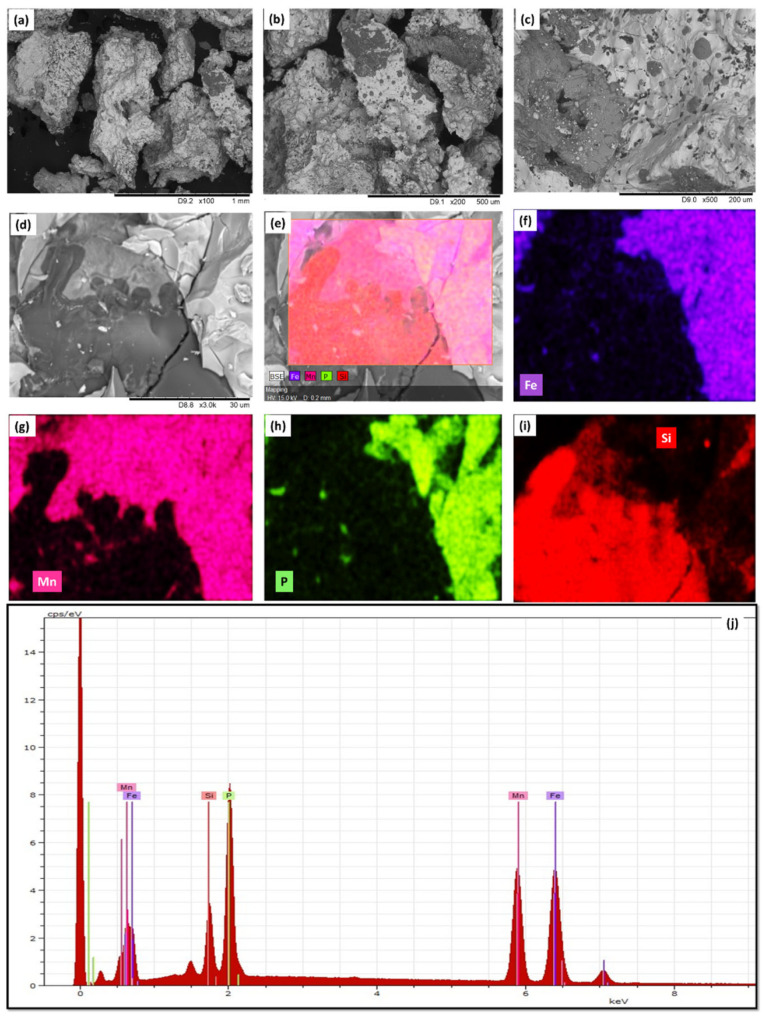
(**a**–**d**) SEM of as-prepared MnFePSi master alloy in bulk form obtained at different magnifications from 1 mm to 30 µm. (**e**–**i**) The EDX elements (Si, Mn, Fe and P) mapping of MnFePSi-master bulk alloy. (**j**) The EDX spectra for one point, illustrating the existence of the Fe, Mn, P and Si elements.

Figure 1 illustrates the morphological features and chemical composition of MnFePSi bulk obtained by SEM/EDX. By analyzing local sections of MnFePSi bulk at different magnifications, we can easily observe that the samples have two main phases (dark and bright spots) with large boundaries (see Figure 1a–d). Figure 1d shows crack is due to the mechanical milling process to prepare powder form for microstructure analysis by using SEM and XRD.

To evaluate the elements’ distribution, EDX/elemental mapping is performed, as shown in Figure 1e–i. From the elemental mapping, we can see the dark spot corresponding to Si-enriched sample regions as compared to the bright from Si-depleted regions. In addition, all elements show inhomogeneous distributions. Therefore, the existence of different microstructural phases can be assumed. More detailed analysis and evaluation of the main microstructural phases will be discussed in the XRD analysis section (Section 3.2). As seen in Figure 1j, the chemical composition for bulk is Mn_40_Fe_32_P_15_Si_15 (_atomic%_)_.

**Figure 2:**
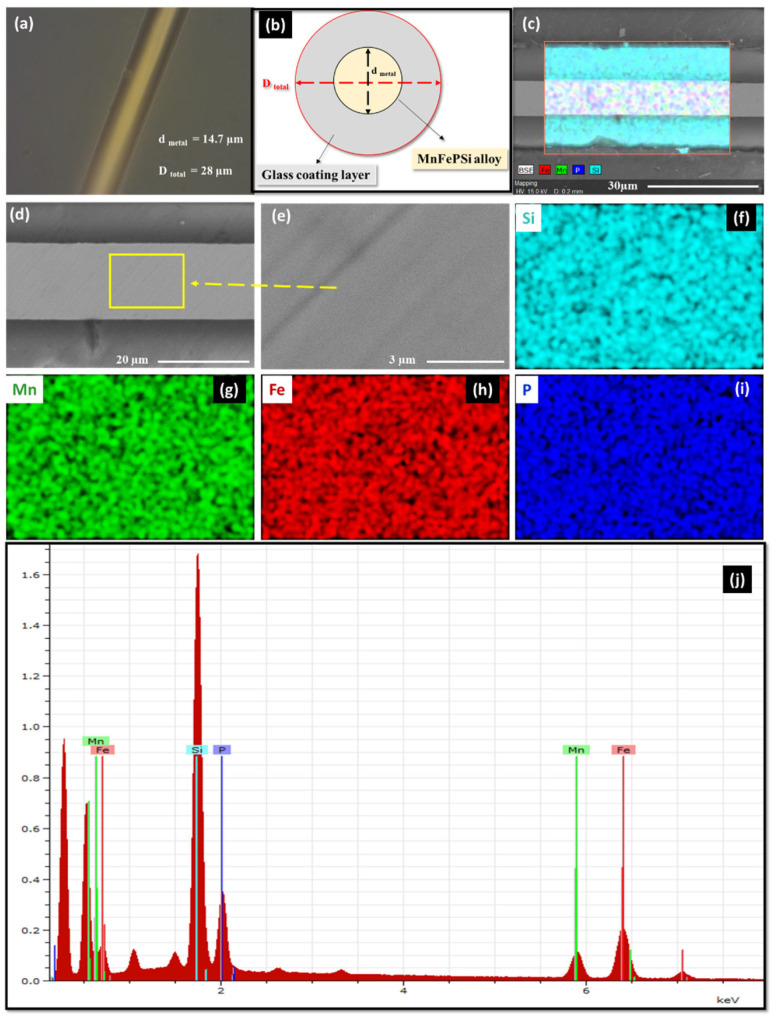
(**a**) The optical microscope image of MnFePSi-glass-coated microwires and (**b**) sketch of MnFePSi-glass-coated microwire. (**c**–**i**) SEM image of MnFePSi-glass-coated microwires and the EDX elements (Si, Mn, Fe and P) mapping; (**j**) the EDX spectra for one point.

For the MnFePSi-GCMWs sample, the morphological and EDX analysis is presented in Figure 2. As seen in Figure 2a–c, the glass coating is visible in a dark color, while the metallic nuclei of MnFePSi alloy appear with bright color. The metallic nucleus looks homogenous. The dark lines seen in Figure 2d,e are due to the polishing process required to remove the glass coating to reveal the microstructural properties of the metallic nucleus. By EDX mapping measurements, a uniform distribution of the Mn, Fe, P and Si elements at the large scale (see Figure 2c) and at the fine scale of the metallic nucleus are observed (Figure 2f–i). The geometrical parameters of the MnFePSi-GCMWs can be extracted from the SEM analysis, which are the diameter of the metallic nucleus, d_metal_ = 14.7 µm, and the total diameter of the microwire is D_total_ = 28 µm, respectively (see Table 1).

For the microwire sample, a small deviation in the chemical composition is observed with a reduced Mn and P content and an increased Si atomic percentage (see Figure 1j and Figure 2j and Table 1). Such changes in the chemical composition of microwires are expected due to the fabrication process of glass-coated microwires involving the melting of the ingot and subsequent rapid melt quenching [36,37,45,48].

Previously, the existence of the interfacial layer (with elevated Si content) between the metallic nucleus and glass coating with a thickness of about 0.5 µm is reported [48].

### 3.2. XRD Analysis of MnFePSi Bulk and Microwire Samples

**Figure 3:**
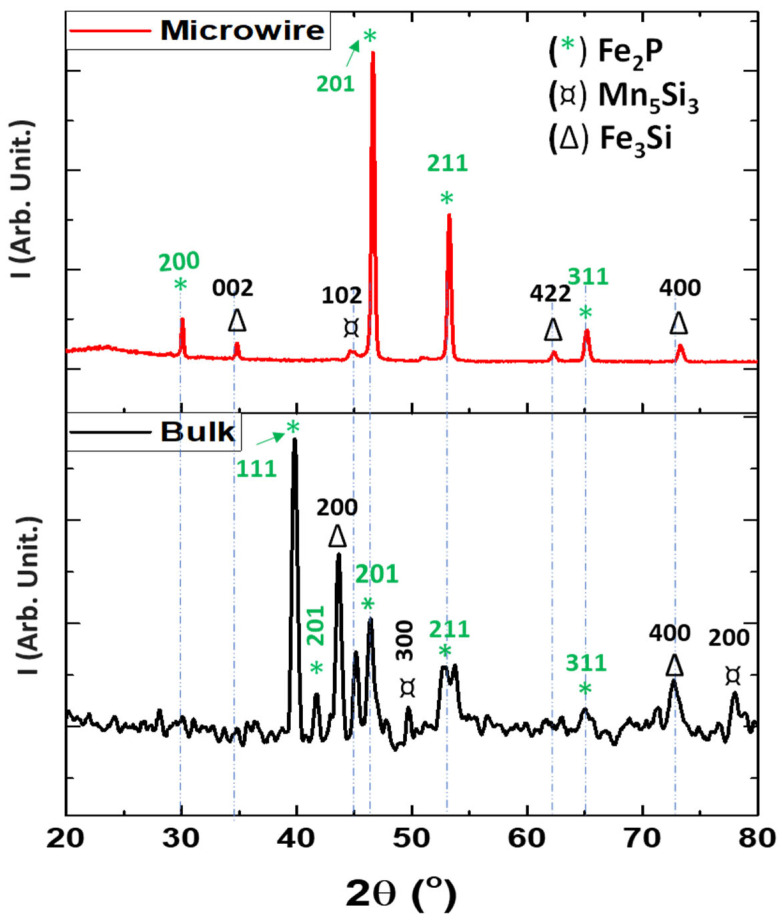
Room temperature X-ray diffraction (XRD) diffractograms of MnFePSi in bulk form (black) and for glass-coated microwire form (red).

XRD analysis was performed at room temperature to determine the crystalline phase differences between the bulk and glass-coated microwire samples. As illustrated in Figure 3, the XRD analysis shows notable differences between the bulk and microwire samples. The MnFePSi bulk and microwire show different phases, where the main phase is hexagonal Fe_2_P (space group P-62m) with a space group with cell parameter a= 5.7981 Å, which nearly agrees with that reported elsewhere [49,50]. In addition, the secondary phases with the hexagonal Mn_5_Si_3_ structure (P63/mcm) and the cubic Fe_3_Si structure (Fm-3m), respectively, are found. As seen in Figure 3, the casting and drawing process involved in preparing the MnFePSi glass-coated microwire affects the microstructure compared to the master bulk alloy, in which the strong peak observed at 2θ = 40°, 41.8° and 43.6° are related to the absence of the Fe_2_P and Fe_3_Si phases. Furthermore, additional peaks at 2θ = 30° and 34.8° are observed. Moreover, a notable reduction in the Mn_5_Si_3_ phase is observed in the microwire sample as compared to the master bulk alloy. These differences in the microstructure and formations of different phases strongly affect the magnetic behavior of the samples as will be discussed in the analysis of magnetic properties. For more information about the average grain size of the corresponding crystalline phase, we used the Debay–Scherrer equation given as follows:

*D_hkl_* = Kλ/(B cos(θ)) (1)

In this equation, *D_hkl_* is the average crystallite size, hkl denotes the Miller indices of the crystal planes, K represents the shape factor (assumed to be 0.9), λ represents the X-ray wavelength (0.154 nm for Cu Kα1 radiation), B denotes the full width at half maximum (FWHM) of the corresponding diffraction peak in radians, and θ represents the Bragg angle.

From the XRD peak’s half-width, the *D_hkl_* value obtained for glass-coated microwires is 36 nm, meanwhile, for the bulk sample, *D_hkl_* is about 24 µm, i.e., the *D_hkl_* value of the bulk sample is almost two orders of magnitude higher than evaluated for the glass-coated microwire sample. The average grain size calculated in the current work for MnFePSi bulk form nearly agrees with that reported elsewhere [19].

### 3.3. Magnetic Properties of MnFePSi Samples

**Figure 4:**
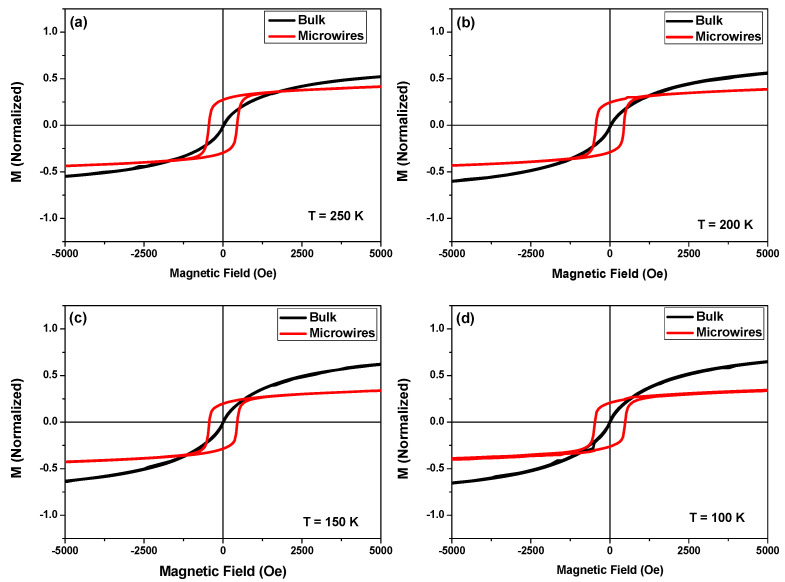
(**a**–**d**) Low field hysteresis loops, measured in magnetic field applied parallel to the axis of microwires in the temperature range from 100 to 250 K for as-prepared MnFePSi-glass-coated microwires (red loops) and master bulk alloy (black loops).

The M-H loops for MnFePSi samples of bulk and glass-coated microwires are measured at a wide range of temperatures (5 K to 250 K). The hysteresis loops are normalized to the corresponding maximum magnetic moments to facilitate a comparative analysis of their magnetic behavior. In Figure 4a–d, selected M-H loops are presented for measuring temperatures between 100 K and 300 K. For measuring temperatures below 100 K, we were not able to obtain regular M-H loops due to the strong contribution of the antiferromagnetic coupling of the Mn_5_Si_3_ phase at low temperature. As shown in Figure 4, notable differences between both M-H loops for MnFePSi-glass-coated microwires and bulk samples are clearly evidenced. The microwire sample appears with harder magnetic properties with coercivity, *H_c_*, near 467 Oe as compared to the bulk sample, which shows rather soft magnetic properties with coercivity of about 5 Oe and vanishing magnetic remanence. Accordingly, *H_c_*, the value for MnFePSi-based glass-coated microwires is almost two orders of magnitude higher than the *H_c_* value observed in bulk samples at the same measuring temperature. The same tendency has been observed for all M-H loops that were measured at low temperatures.

Compared to other studied MnFePSi samples (ribbons, bulk and thin films), the relatively hard magnetic properties appear unusual, since MnFePSi alloys typically exhibit rather soft magnetic behavior below room temperature. The microstructure and chemical composition of MnFePSi alloys have a significant impact on their magnetic characteristics. The enhanced *H_c_* values, obtained in MnFePSi-based glass-coated microwires, can be attributed either to higher internal stresses associated with the glass-coating layer, induced during the manufacturing process or to a distinct microstructure compared to other MnFePSi samples (see XRD section). Due to the glass having low heat conductivity, the glass coating not only causes high internal stresses, but also has an impact on the quenching rate and, hence, can modify the physical properties and structure of the metallic nuclei.

For a deeper understanding of the magnetic behavior of MnFePSi samples, the magnetization vs. temperature (M vs. T) measurements at the different magnetic fields have been carried out. Due to the limitations of the PPMS setup, the temperature range is from 400 to 5 K. To ensure a more accurate comparison of the magnetic properties between the bulk and glass-coated microwire samples, we normalized the magnetization data for all measurements. This normalization involved dividing the magnetization value at each temperature by the maximum magnetization measured at 5 K (M/M_5K_). Magnetic saturation can be challenging to determine precisely in these composite microwires due to their mixed material composition. Even small errors in calculating saturation could lead to misinterpretations of the key differences between the two sample types. This normalization approach minimizes the impact of such errors and allows us to focus on the true variations in magnetic behavior between the annealed and as-prepared states.

The performed analysis is focused on three different protocols, zero-field-cooling protocol, ZFC, and field-cooling protocol, FC, where the samples are cooled from 400 K to the lowest point, i.e., 5 K with different applied external magnetic fields from 1 to 20 kOe. For the field heating protocol, FH, the reversed process has been conducted at the same applied external magnetic field. For the MnFePSi-glass-coated microwires, the magnetic measurements are performed parallel to the wire axis where the easy magnetization axis is expected.

**Figure 5:**
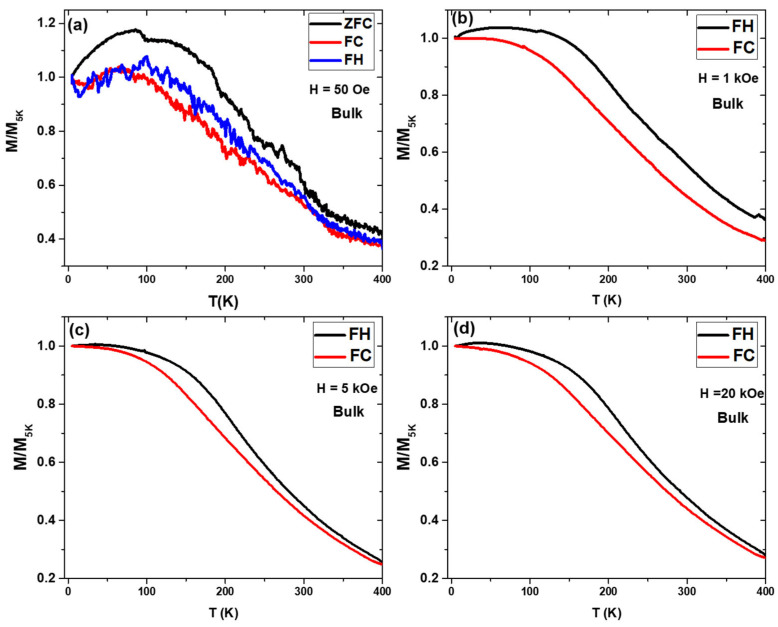
(**a**) ZFC, FC and FH curves measured at 50 Oe of MnFePSi bulk at temperature range of 400 K to 5 K. (**b**–**d**) FC and FH with different applied magnetic field H = 1 kOe, 5 kOe and 20 kOe, respectively.

The ZFC, FC and FH curves for bulk MnFePSi samples are presented in Figure 5. From the ZFC, FC and FH magnetization curves, it seems that the Curie point where the transition from ferromagnetic state to paramagnetic state is over 400 K. As reported in the previous works, the transition temperature for MnFePSi alloys can vary from 467 K to 100 K depending on the chemical compositions, annealing condition, secondary phases and fabrication-processing techniques [15,16,17,18,19,20,21,22,23,24,25,26,27]. ZFC, FC, and FH curves measured at a low magnetic field (H = 50 Oe) show a weak signal, where the curves appear very nosey. In addition, notable separation between the curves is seen with irreversible magnetic behavior below T = 100 K (see Figure 5a). For FC and FH magnetization curves, measured at H = 1 kOe, 5 kOe and 20 kOe notable ferromagnetic ordering is seen for the temperature range from 400 K to 5 K, where M/M5K has monotonic increasing by decreasing the temperature from 400 to 5 K (see Figure 5b–d). In addition, the highest separation between the FH and FC curves is observed at the applied external magnetic field at 1 kOe (Figure 5b). However, the curves measured at H = 5 kOe and 20 kOe show almost similar magnetic behavior.

The main reason for such kind of mismatching between FC and FH curves can be related to the existence of different phases as described in XRD analysis. It seems that the antiferromagnetic state stability of the Mn_5_Si_3_ phase is limited by the applied external magnetic field, where dominating ferromagnetic behavior is detected at all measuring temperatures range, i.e., 5–400 K at H = 1 kOe, 5 kOe, and H = 20 kOe. Thus, the existence of Fe_2_P and Fe_3_Si phases has a strong impact on the temperature dependence of magnetic properties of MnFePSi alloy at applied high magnetic field ≥ 1 kOe and causes the separation between the FH and FC magnetization curves.

**Figure 6:**
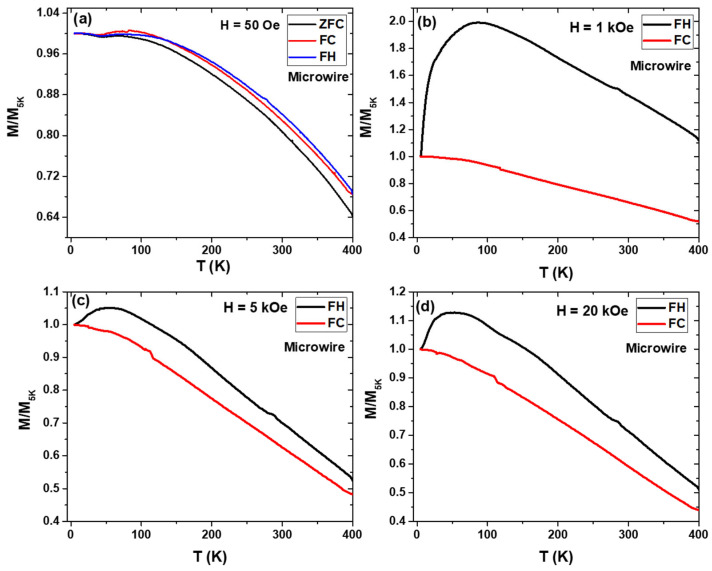
(**a**) ZFC, FC and FH curves measured in MnFePSi-glass-coated microwire at 50 Oe in the temperature range of 400 K to 5 K. (**b**)–(**d**) FC and FH with different applied magnetic field H = 1 kOe, 5 kOe and 20 kOe, respectively.

We measured the M/M_5K_ vs. T dependencies for MnFePSi-glass-coated microwire using the same conditions as for the master bulk alloy. The ZFC, FC and FH curves measured at H = 50 Oe show completely different behavior compared to the bulk alloy curves. First, the curves show negative M/M_5K_ values due to the high coercivity (Hc ≈ 467 Oe), and the positive values of M/M_5K_ shown in Figure 6a were obtained after correction by multiplication (−1). The absence of noise in the magnetic signal for the microwire sample must be attributed to the enhancement of the magnetic response of MnFePSi-GCMWs for the low magnetic field compared to the bulk alloy.

As we can see in Figure 6b, distinct magnetic behavior is observed for MnFePSi-based glass-coated microwires, as compared to the bulk sample. Notable irreversibility is observed for FH magnetic curves at H-values of 1 kOe, 5 kOe and 20 kOe. The existence of the irreversible magnetic behavior in MnFePSi-glass-coated microwires can be attributed to several possibilities, such as the different magnetic signal impurities that come from the existence of different magnetic phases (ferromagnetic Fe_2_P, Fe_3_Si) and antiferromagnetic (Mn_5_Si_3_). The dominant different phases of Mn enhance the strength of the antiferromagnetic coupling below 100 K. Thus, the MnFePSi-based glass-coated microwires show large irreversibility in magnetic behavior at a wide range of applied external magnetic fields. In addition, observed differences in phase composition can produce different magnetic responses with temperature and the applied external magnetic field. This can produce different degrees of mismatching between the FC and FH magnetization curves above and below 100 K. Such irreversible behavior has been reported previously in Heusler alloys-based glass-coated microwires (particularly, in Heusler-based Mn alloys) [31,33,36,45].

Finally, the change in the stress induced by the covering glass layer with the temperature can affect M(T) dependencies, producing a gradual change in the magnetic microstructure of the metallic nucleus and, hence, affecting their magnetic response with temperature and magnetic field [30,36,42,43,44].

On the other hand, rapid quenching from the melt (involved in the fabrication of glass-coated microwires) can affect the fine structure (average grain size) and, hence, affect both coercivity and M(T) dependencies [9]. Additionally, it is commonly recognized that the crystallization process involving atomic diffusion is affected by the presence of mechanical stresses [51]. Thus, experimentally, it was demonstrated that the magnetic properties [52], the structure and even precipitation of the crystalline phases upon annealing of Heusler-type microwires are affected by the mechanical stresses induced by the glass coating [53].

As described above, almost two orders lower average grain size (36 nm) value is obtained for MnFePSi-based glass-coated microwires as compared to *D_hkl_* ≈ 24 µm, as estimated in the bulk MnFePSi sample. Definitively, such a substantial difference in *D_hkl_* plays an important role in the rather different magnetic behavior of MnFePSi-based glass-coated microwires.

Manufacturing glass-coated microwires by using the Taylor–Ulitovsky method involves rapid solidification of the materials with substantially different thermal expansion coefficients [9,29,30,44]. This huge difference in thermal expansion coefficients between the metallic alloy and glass leads to significant internal stresses within the microwires [9,36,37]. These stresses are distributed in a complex way within the metallic nucleus [9]. Extensive theoretical calculations and indirect experiments suggest that the axial internal stresses are the highest within the metallic nucleus of the microwire [42,44]. Estimated values of internal stress are between 100 and 1000 MPa [9,37,39]. In comparison, the stress exerted by a magnetic field in bulk Heusler alloys with large magnetic field-induced strain lies between 1 and 7 MPa, highlighting the significantly larger impact of the glass coating on internal stress levels. These substantial internal stresses, induced by the mismatch in thermal expansion coefficients of metallic alloy and glass coating, represent a major obstacle to achieving the martensitic transformation in glass-coated microwires prepared from Heusler alloys [32,54]. This martensitic transition allows the material to exhibit a substantial magnetocaloric effect and magnetic shape memory effect in Heusler-type microwires. Therefore, relaxing these internal stresses becomes a crucial step in harnessing the full potential of glass-coated microwires. Various strategies can be employed for stress relaxation, ranging from annealing treatments to mechanical modification of the glass coating composition. Implementing such methods would pave the way for unlocking the exciting applications of these innovative materials. However, in the current study, we were not able to obtain the first-order phase transition, where the Curie points for samples (microwires and bulk form) are over 400 K. Additionally, the first-ordered transition for MnFePSi alloys is very sensitive to many parameters, such as the chemical composition, the fabrication process, the microstructures and the phases. However, the current investigation reveals the potential of the Taylor–Ulitovsky method to modify and tailor the magneto-structural properties of the metallic core alloys compared to its bulk master alloy and to produce low dimensional materials by single-step fabrication technique.

## 4. Conclusions

The fabrication and characterization of MnFePSi-based glass-coated microwires by using the Taylor–Ulitovsky method is reported for the first time. Extensive investigation on the structure, morphological and magnetic properties at different magnetic fields and temperatures has been carried out. The difference in the microstructure properties of MnFePSi-glass-coated microwires can be related either to the internal stress or different microstructure affected by the rapid melt quenching involved in the fabrication process of glass-coated microwires. A remarkable difference in average grain size from 24 µm for bulk MnFePSi to 36 nm for the glass-coated microwire sample is observed. The M-H loops of MnFePSi-glass-coated microwires measured at different temperatures show enhanced coercivity value compared to the bulk sample, being almost two orders of magnitude higher. In addition, an interesting irreversibility magnetic behavior is observed in MnFePSi-glass-coated microwires, which can be well controlled by using a wide range of external magnetic fields. The provided study reveals the suitability of the Taylor–Ulitovsky method to tailor and change the physical properties of hosting metallic nuclei compared to its master alloy. Further investigations are needed to study different parameters such as the effect of the annealing and geometrical aspect ratios on the magneto-structural and thermomagnetic behavior of MnFePSi-based glass coating microwires.

## Figures and Tables

**Table 1:** Chemical compositions and microstructure parameters MnFePSi alloy in bulk and glass-coated microwire forms.

Sample	Chemical Composition	D_total_	d_metal_	Average Grain Size	Phases
A—Bulk	Mn_40±2_Fe_32±3_P_15±3_Si_15±2_	-	-	24 µm	Fe_2_P, Mn_5_Si_3_, and Fe_3_Si
B—Microwires	Mn_38±3_Fe_30±2_P_13±2_Si_19±3_	28 µm	14.7 µm	36 nm	

## Data Availability

Data are contained within the article.
